# Supplementary material for: Are carbon emissions trading and green financial instruments synergistic? -Comprehensive quantitative research based on content analysis
Source: PLoS One. 2024 Mar 7;19(3):e0298601. doi: 10.1371/journal.pone.0298601 (PMC10919601; doi:10.1371/journal.pone.0298601)
Supplement: S1 Data — (ZIP) [file pone.0298601.s001.zip › Data/地级市Z_F工作报告.zip!/╜¡╬≈/─╧▓2╩╨/└┤╫╘╣1⁄2╓┌║┼í╛┬φ┐╦╩2╛▌═°í┐_2015.docx]

各位代表：

现在，我代表南昌市人民政府，向大会作工作报告，请予审议，并请各位市政协委员和列席会议的同志提出意见。

一、2014年工作回顾

2014年，全市上下在省委、省政府和市委的正确领导下，坚决贯彻“发展升级、小康提速、绿色崛起、实干兴赣”十六字方针，以打造核心增长极为主线，以全面深化改革为动力，以践行群众路线为保障，统筹抓好稳增长、促改革、调结构、惠民生各项工作，经济社会保持持续健康较快发展。全年实现地区生产总值3667.96亿元，完成年度计划99.7%，增长9.8%；完成财政总收入638.3亿元，完成年度计划100.8%，按省口径计算增长16.2%；地方一般公共预算收入342.2亿元，完成年度计划103.3%，增长17.2%；规模以上工业增加值1380.6亿元，完成年度计划105.1%，增长11.9%；500万元以上固定资产投资3434.25亿元，完成年度计划98%，增长18.6%；社会消费品零售总额1429.21亿元，完成年度计划99%，增长12.5%；海关出口总额84.17亿美元，完成年度计划109.6%，增长15.2%；实际利用外资32.14亿美元，完成年度计划100.4%，增长8.42%；城镇居民人均可支配收入29091元，完成年度计划100.7%，增长10%；农村居民人均可支配收入12414元，完成年度计划104%，增长11%。万元生产总值能耗下降2.5%，完成年度计划100%。城镇就业率达到95%，居民消费价格指数为2.5%。

一年来，主要做了以下工作：

（一）大力实施四个“三年强攻计划”，打造核心增长极已经起势

一是打造核心增长极的产业支撑明显增强。工业经济增势强劲。完成工业投资1397.46亿元，规上工业主营业务收入突破5000亿元大关、增加值达到1380.6亿元。产业集群扬优成势。四个千亿产业快速发展，其中，食品产业主营业务收入突破千亿，电子信息产业连年大幅增长。重点企业持续发力。江铃集团、正邦科技、欧菲光主营业务收入分别突破500亿、200亿、100亿元大关。工业园区扩容升级。南昌高新区、南昌经开区、小蓝经开区三大国家级园区快速壮大。服务业发展态势良好。第三产业增加值占生产总值比重突破40%，达到40.5%。商贸、金融、旅游、会展、文化创意等产业快速增长，全省金融商务区累计入驻各类金融机构131家；旅游接待总人次和旅游综合收入保持高速增长；成功举办世界低碳大会、中国智博会等全国品牌展会。电子商务产业快速发展，“一基地多园区”产业格局基本形成；绳金塔夜市等一批特色商业街、夜市悄然兴起。农业经济稳步发展。粮食总产244.97万吨，实现“十一连丰”。市级以上农业产业化龙头企业456家；溪霞国家农业综合开发现代农业园区建设、“一村一品”强村富民工程等有效推进。

二是打造核心增长极的空间支撑明显增强。积极推动大建设。九龙湖万达文化旅游城、绿地国际博览城项目加快推进，片区路网工程完成形象进度超过90%，南昌西站基本建成，九龙湖湖体综合整治和隧道工程加快实施，综合性住房小区等还建房建设全面铺开。敢于面对“旧”难题。启动“旧改棚改”项目97个，完成改造33840户，征迁改造面积约685.884万平米；两轮“拆违拆临”专项行动共拆除面积38万平米；深入开展土地收储和闲置土地清理工作，储备土地17万亩。基础设施更完善。地铁1号线预计今年年底建成，2号线加快建设，3号线前期工作进展顺利；朝阳大桥、九洲高架、象湖隧道、红谷隧道、昌栗、昌宁高速等项目加快推进。

三是打造核心增长极的特色支撑明显增强。县域经济活力迸发。四县一区完成财政总收入155.9亿元，占全市24.4%。四县一区县域主导产业逐步壮大。安义县铝型材销售占全国市场份额70%；进贤医疗器械产业比重占全省的70%,一次性输液器产品占国内市场份额的31%；南昌县在全国百强县排名前移15位。非公经济蓬勃发展。实现增加值2126.65亿元，占全市GDP58.3%；完成固定资产投资2599.02亿元，占全市总量75.7%。

（二）始终坚持深化改革扩大开放，发展动力更加强劲

一是创新发展动力增强。重点领域改革集中突破。大幅放宽落户条件，推进城区“一元化”户口登记，户籍制度改革走在全国省会城市前列；强力推进第九轮行政审批制度改革，截至目前，共保留市本级行政审批事项231项；在全省率先推进农村土地承包经营权流转交易市场建设试点工作，服务范围基本实现全覆盖；推进开发区干部人事制度改革，科学高效的选人、用人机制初步建立；市级公费医疗改革顺利推进。关键环节改革大胆探索。投融资体制改革深入推进，有效整合六大国有投融资平台，全面探索“1+6+X”模式，引进风险投资管理机构，设立了洪城资本创业投资、临空创新股权投资、联昌基金等三个产业投资基金，“财园信贷通”成效明显。

二是开放型经济加快升级。招商引资结硕果。组织参加了一系列重大招商活动，签约重大项目224个，签约投资总额1631.2亿元。项目攻坚见成效。深入开展“产业项目攻坚突破年”活动，落户重大项目172个，投资总额946.9亿元；开工重大项目144个，投产71个，欧菲光三期、海派通讯、鸿利光电、恒动汽车电池、华南城（电商）等一批重大项目落户，并开工建设。对外贸易有突破。进出口总额突破百亿美元大关,达到122.3亿美元，同比增长25.9%，占全省总量28.54%。 企业加快“走出去”。新增境外投资企业25家，中方协议投资额5.91亿美元。航空口岸更开放。加密和新开辟了一批航班航线，经营航线达79条，旅客吞吐量724万人次，增长6.3%；货邮吞吐量4.61万吨，增长14.1% 。

三是抱团发展态势良好。 “内联”合作不断升级。深入推进昌九一体化，《昌九一体化发展规划（2013-2020）》编制完成，与九江部分实现了金融、电信、社保接续等公共服务的同城化，昌九大道等城际基础设施加快建设。昌抚一体化步伐加快。丰樟高、鄱余万加快融入南昌，南昌一小时经济圈加快构建。“外融”对接初见成效。主动融入长江经济带，与武汉、长沙、合肥签订了《长沙宣言》，长江中游城市群省会城市合作长效机制基本建立，基础设施、产业发展互联互通，市场一体化步伐加快。加快融入闽东南和泛珠三角区。

（三）突出优化自然社会政治生态，发展环境明显改善

一是自然生态更加优美。深入开展“蓝天、清流、净土”行动，城乡环境进一步改善。天更蓝。建筑扬尘、汽车尾气污染专项整治和打击违法排污企业专项行动深入开展，新禁燃区锅炉拆改全面推进，推广新能源汽车459辆，区域大气污染联防联控成效显著，空气质量优良天数294天，在第一批实施新标准74个城市中排第25名，同比前移10位，优良率80.5%，同比提高19.7个百分点。水更绿。全面推进水环境综合治理，象湖、抚河截污工程基本完成，梅湖、玉带河截污工程正式启动，前湖、乌沙河、幸福渠流域综合整治工程和军山湖、幸福水库、溪霞水库畜禽养殖污染防治深入推进；农村水环境综合整治试点工作和畜禽禁养区专项整治行动启动实施。全市主要河流水质达标率达93%，集中式饮用水源地水质达标率100%。土地更干净。加快推进土壤信息库建设，大力开展土壤污染试点调查，突出做好进贤文港镇重金属集控区建设、重金属防控区土壤修复试点等各项防治工作。农村环境更美好。大力实施特色小镇、“五位一体”综合示范村、新农村点整治建设，开展了第二批主要通道沿线“五化”综合整治、村民建房规划建设管理综合整治、农村清洁工程和“六好农户、六好村庄”创建等工作。

二是社会文明程度稳步提升。“创文保卫”成效明显。各类专项整治全面推进，卫生死角、垃圾广告顽疾得到有效治理，《南昌市城市建筑垃圾管理条例》颁布实施,餐厨垃圾统一收集、处理全面启动。市民对城市环境面貌改善满意率达85.7%，中央文明办复审组、国家卫生城市复审组对我市“创文保卫”工作给予充分肯定。城市管理愈发精细。着力构建“两级政府、三级管理、四级网络”城市管理体制，在56个街道（镇、管理处）、420个社区建立了城市管理综合执法队伍和社区城市管理工作站。环卫作业全面市场化，生活垃圾无害化处理率100%，道路机械化清扫、冲洗率提高到60%以上。红谷滩智慧城市建设取得阶段性成果。

三是政务环境明显好转。群众路线教育实践活动扎实开展。市政府党组6个方面整改任务和14个方面专项整治任务全面完成，建立健全24项制度，“四风”突出问题得到有效整治，政府系统作风建设取得新进步。法治政府建设步伐加快。深入推进“六五”普法，坚持领导干部学法制度和政府法律顾问制度，开展规范性文件和重大行政执法决定备案审查与清理工作，建立法治政府建设指标体系。推进集中行使行政复议权试点工作，建立市县乡三级行政调解组织网络体系，信访工作依法有序推进。自觉接受人大及其常委会的法律监督、工作监督和政协的民主监督，全年共办理人大代表建议、政协提案533件，办复率100%。全年主动公开政府信息217661条，政府行政更加透明。深入开展社会治安突出问题严打整治等专项行动，治安防控体系建设大力推进，安全生产形势总体平稳，社会保持和谐稳定。认真落实党风廉政建设责任制，严格遵守中央八项规定，廉洁政府建设进一步加强。

（四）着力发展社会事业，人民生活水平稳步提升

全市教育、社会保障和就业、医疗卫生与计划生育、住房保障等关系民生的支出共191亿元，增长14.1%，占全市地方一般公共预算支出的40.3%，有效保障了民生需求。

一是社会事业统筹推进。学前教育三年建设行动计划深入实施，新建公办幼儿园466所，增加学位43875个。农村义务教育学校标准化和城区义务教育学校网点建设持续推进。学区化管理试点范围扩大，实现城区义务教育“零择校”。开展优质普高和一般普高校际共建试点。教育关爱工程全面推进，安排25535名外来人员随迁子女在我市公办义务教育学校起始年级就读，农村留守儿童入学率达100%，资助家庭经济困难学生22787人。举办了2014年江西省首届文化活动周，推进了市群艺馆新馆建设、南昌美术馆改造升级、市强制隔离戒毒所改扩建工程，更新农家书屋出版物679家。县级公立医院综合改革深入推进，三个试点县取消药品加成让利近6000万元。全民健身活动深入开展，镇村体育设施更加健全。

二是民生实事如期完成。全市新增城镇就业7.9万人，城镇“零就业家庭”安置率100%；新增转移农村劳动力4.9万人；新增发放小额担保贷款11.15亿元。“五险”参保任务超额完成。城区“三个中心”建设扎实推进。城乡低保标准、分散供养标准进一步提高，为1.338万名残疾人提供康复救助与服务，办理法律援助案件、事项2.75万件。中心城区规划建设了16个棚户区改造安置房建设项目，已开工1.6万套；基本建成9561套保障性住房，发放廉租住房租赁补贴10215户，5900套公租房实施配租。推进了7.18万贫困人口、100个扶贫村“精准扶贫”工作。全面解决14.7万农村居民和4.8万农村学校师生饮水安全问题。新建和升级改造37家农贸市场，巩固提高50家社区菜店和100余家平价商店。

与此同时，国防建设、人民防空、拥军优属、国家安全、综治维稳、民族宗教、对台事务、外事侨务等工作继续加强；审计统计、工商税务、食品药品、粮食安全、质监安监、参事文史、社科研究、档案史志、地名水文、科普气象、老龄慈善、工会、青少年、妇女儿童、防灾减灾、爱国卫生、红十字会、机关事务、消费者权益保护等工作取得喜人成绩。

各位代表!这些成绩的取得，是省委、省政府和市委正确领导的结果，是人大、政协监督支持的结果，是全市上下共同努力的结果。在此，我代表市人民政府向全市人民，向各民主党派、工商联、人民团体和各界人士，向驻昌部队、武警官兵和政法干警，向所有关心支持南昌发展的朋友们，表示诚挚的感谢和崇高的敬意!

看到成绩的同时，我们也清醒地看到存在的困难和问题，主要表现在：发展速度不快、环境不优、动力不足的问题仍然存在；城市规划、建设、管理还存在薄弱环节；制约经济社会发展的体制机制障碍尚未完全革除，政府服务和干部作风有待进一步提高，执行落实不力、办事效率不高的问题依然突出。对此，我们高度重视，将采取切实有效措施加以解决。

二、2015年工作思路和主要举措

2015年是全面深化改革的关键之年，是全面推进依法治市的开局之年，是全面完成“十二五”规划的收官之年，也是全面打造核心增长极的提升之年。我国经济长期向好的基本面没有改变，我省加快发展的重要战略机遇期没有改变，我市打造核心增长极的向上势头没有改变。我们既要看到各种不利因素和风险挑战，又要看到积极因素和有利条件，坚定发展信心，克服艰难险阻，抢抓战略机遇，务实奋进做好各项工作。

今年政府工作的总体要求是：认真贯彻落实党的十八大和十八届三中、四中全会、中央经济工作会议以及省委十三届十次全会、市委十届十次全会精神，以全面打造核心增长极为主线，以全面深化改革为动力，以全面推进依法治市为保障，以全面优化发展环境为总抓手，坚定信心，增强定力，立说立行，真抓实干，努力保持经济社会持续健康较快发展，为全省实现“五年决战同步小康”的目标作出南昌应有贡献。

今年经济社会发展的主要预期目标是：

地区生产总值增长10%左右；

财政总收入增长12.5%；

地方一般公共预算收入增长12.5%；

全社会固定资产投资增长20%；

规模以上工业增加值增长12%左右；

社会消费品零售总额增长12.5%；

利用外资实际到位资金增长10%；

实际利用内资增长8%；

海关出口总额增长5%；

城镇居民人均可支配收入增长10%；

农村居民人均可支配收入增长10%；

万元生产总值能耗下降2%；

人口自然增长率控制在8‰以内；

居民消费价格总水平涨幅控制在3.5%以内；

完成国家下达的节能减排计划任务。

把握好以下原则：

一是正确处理速度与质量的关系。牢牢把握稳中求进工作总基调，巩固“稳”的基础，保持“进”的势头，提高“进”的质量，更加注重资源配置效率、产业结构升级和生态环境保护。

二是正确处理改革与法治的关系。坚持问题导向，不抢跑，不拖宕，深入推进各项改革；坚持法治思维，使各项改革于法有据，真正实现改革与法治相辅相成、相互促进。

三是正确处理政府与市场的关系。坚持市场配置资源的决定性作用，更好发挥政府作用，对私权法无禁止皆可为，对公权法无授权不可为，对单位和干部法定职责必须为，不断提高政府治理能力和治理水平。

四是正确处理工业化与城镇化的关系。发挥城市快速集聚产业和人口的作用，确保城镇化有产业带动、工业化有城镇支撑，加快“两化融合”发展步伐。

五是正确处理发展与民生的关系。牢固树立发展为要、民生为本的理念，进一步加大民生投入，不断促进公共服务均等化，使发展成果更多惠及人民。

今年，我们将突出抓好以下工作。

（一）主动适应经济发展新常态，推动经济发展提质增效

更高水平发挥“三驾马车”稳定增长的作用，加快推动产业升级步伐，全面激发经济发展活力，努力在经济新常态下实现“调速不减势、量增质更优”。

一是全力促进经济稳定增长。稳定投资需求。围绕对接国家“七大工程包”和省里“八大工程包”，突出抓好航空工业城、北斗卫星产业园、鸿利光电产业基地、欧菲光液晶显示模组、济民可信等重点工业项目建设，突出抓好万达文化旅游城、南昌国际博览城、华南城（电商）物流园等重大服务业项目建设，突出抓好南昌市民中心、南昌外国语学校九龙湖校区、省皮肤病医院新院、南昌市博物馆等重大社会事业项目建设。挖掘消费潜力。千方百计提高中低收入居民收入，努力提高消费能力，让百姓“能够消费”。规范引导企业利用店庆、传统节假日，安全有序开展各类展会和促销活动，大力促进节假日和会展消费，让百姓“乐于消费”。有效稳定住房、汽车等大宗商品消费，促进消费升级。促进现代物流发展，不断完善消费环境，让百姓“方便消费”。防范金融风险。理顺政府债务管理体制，防范化解企业债务风险，严格规范民间融资，严厉打击非法集资，确保金融不出现区域性系统风险。

二是着力推动产业结构优化升级。发展升级的核心是产业升级，关键是转方式、调结构。强化产业攻坚。始终坚持“二三并举、双轮驱动”，推动产业向中高端发展。继续实施工业三年强攻计划，力争工业增加值突破1500亿元；新增千亿园区1个、百亿企业2户、50亿企业2户。集中培育壮大战略性新兴产业，不断做强做优工业经济。继续实施服务业三年强攻计划，大力引进和建设一批服务业重大项目，培育一批龙头企业，努力提升服务业占国民经济比重。充分发挥现代农业“接二连三”效应，努力实现全市农业招大引强有所突破、龙头企业升级有所突破、打造南昌农业品牌有所突破，推进农业产业化转型升级。聚焦园区战场。以三大国家级开发区和临空经济区为主战场，为工业发展提供有力平台。重点发展高新区生物医药、航空和光电产业，经开区机电和电子信息产业，小蓝经开区汽车及零配件、食品加工产业，临空经济区电子信息和现代物流产业。力争3个国家级开发区主营业务收入全过千亿，1个省级工业园区突破500亿。推动集群发展。工业方面主要是继续打造4大千亿和5大五百亿产业集群。加快建设电子商务、服务外包、金融、物流、商贸、商务会展、文化创意、旅游休闲、餐饮、新型专业市场等十二类服务业集聚区，全力建设全省金融商务区，力争各类金融机构（企业）达到160家。突出抓好现代农业示范园区建设，力争规模达到70万亩。

三是积极培育经济新增长点。以更“活”的市场、更“实”的创新、更“好”的政策，为南昌发展挖掘培育更多新的经济增长点。发展互联网经济新业态。积极推动以信息技术为基础的新业态发展，研究制定“互联网+”行动计划。依托各类平台，结合电子商务进乡镇、进农村工作，多措并举发展电子商务，力争全市新增交易额过10亿元的电商3家，过5亿元的电商5家。积极发挥江西列入国家物联网重大应用示范工程区域试点作用，实施好双胞胎集团、正邦科技、泰豪软件等相关企业的物联网技术应用示范工作。培育消费新热点。顺应旅游休闲、健康养老、教育医疗、体育健身发展新趋势，深入实施旅游强市战略，使南昌成为中部乃至全国重要的旅游目的地；加快培植健康和养老服务产业，推动健康服务业医养一体化。推动大众创业。大力支持民营经济发展和中小微企业发展。放宽民间投资准入领域，进一步消除民营资本进入的隐性壁垒，充分发挥民间资本作用。加大全民创业支持力度，最大限度简化小额贷款反担保程序，延长担保责任期限，逐步建立高校毕业生创业小额担保贷款风险核销机制。全力打造南昌市华南城大学生电子商务创业孵化基地；深入推进“创业孵化100”、 “加速成长100”和“信用促进100”工程，打造“小微企业创业孵化岛”，不断掀起全民创新创业的新高潮。

四是开创开放型经济新格局。坚持“全域开放”理念，全方位推进对外交流合作。融入“一体化”格局。以临空经济区为战略支点，争取设立综合保税区，集中力量加快推进昌九一体化，规划推进昌抚合作示范区建设。以基础设施互联互通、产业发展互补互促、市场体系自由一体为重点，积极对接“一带一路”和长江经济带国家战略，有序融入全球经济一体化格局，全面分享“一带一路”国家战略带来的“中国世纪”红利。提升“引进来”实效。务实推进产业发展协调决策推进机制，完善重大项目绿色通道、跟踪调度、督查制度。围绕“4大千亿、5大五百亿”产业，着力推进央企、外企、民企“三维”对接，大力开展产业项目招商攻坚和补链、强链招商，签约和落地一批体量大、业态好、后劲强的产业项目。加快“走出去”步伐。大胆鼓励有条件的企业“走出去”，抢占更多国际市场份额。以“靠近原材料供应、靠近产品销售市场”为导向，鼓励境外投资办厂。鼓励和引导战略性新兴产业开展对外投资与经济合作，提高国际化发展水平。

（二）主动适应全面深化改革新常态，以改革创新增“红利”强动力

坚定不移推进改革创新，进一步破除影响发展的体制机制障碍，释放改革新红利，激发发展新活力，赢得竞争新优势。

一是全面深化重点领域改革。深化行政管理体制改革。抓好第九轮行政审批制度改革和县区机构改革，大力推进“三单一网”改革试点。探索开发区体制机制创新，建立健全开发区差异化考核指标体系。全面深化财税体制改革。进一步完善财政体制，严格财政预算；适时推进存量非住宅计税价格评估；积极争取环境保护“费改税”先行试点。深入推进投融资体制改革。稳步推进投融资平台分类改革。加强地方政府自主发债工作，积极争取发行各类专项债券。鼓励各类企业通过发行公司债、企业债等方式拓宽融资渠道。探索发展产权交易市场，研究促进碳排放权交易平台建设。积极探索PPP模式，依法依规放宽社会资本进入领域、确立公私合营项目，建立健全社会资本盈利和退出机制。探索推进混合所有制经济发展。启动新一轮市属国有企业改革，吸引各类资本参与国有企业的改制重组，组建一大批适应市场的混合所有制企业。推进国有资本、集体资本、非公有资本等相互参股控股、合资合作，大力发展混合所有制经济。全面深化土地和农村体制改革。改革工业用地供应方式，探索建立城乡统一的建设用地市场。建立不动产统一登记制度，全面完成农村土地承包经营权确权登记颁证工作，加快农村土地承包经营权流转。开展农村集体经济组织产权制度改革试点，做好林垦企业改革扫尾工作。推进事业单位分类改革。积极探索事业单位管办分离，继续推进事业单位法人治理结构试点。推进政府购买服务改革。坚持公开公正、廉洁透明、降低成本、提高时效的原则，研究制定政府购买服务的实施办法，提高政府服务市场化保障水平。强化市场监管创新。完善市场监管体系，重点推进企业监管改革试点，构建科学高效的事中事后监管模式，依法规范生产、经营、交易等市场行为，大力营造诚信市场环境。深化户籍制度改革。开展农业转移人口市民化成本分担机制改革试点。探索实行全市范围内居民户口通迁制度。统筹考虑社会保障和公共服务配套等因素，适当降低落户门槛，合理确定落户条件，保持人口适量增长。

二是深入实施创新驱动战略。强化体制机制创新。围绕我市重点产业领域，加快推行科技成果产权制度改革、股权和分红激励等政策，不断推动产学研结合和协同创新。强化科技创新。推进高新区按照国家自主创新示范区标准，发展成为省市创新发展的核心引领区；聚焦扶持一批重大科研项目和科技成果产业化项目，培育一批科技“小巨人”企业，鼓励和推动科技孵化器等创新创业平台建设。支持小微企业特别是科技型企业开展基础创新融资，鼓励社会融资担保机构开展科技担保业务。强化管理创新。创新财政资金尤其是三个产业专项引导资金管理，改变过去“撒胡椒面”的方式，用专业说话，按程序办事，集中资金扶持引导重点产业、重点项目。积极推进企业管理创新，重点是创新企业管理人员薪酬体系和人才激励机制，探索股权、期权激励机制。强化服务创新。深入开展“暖企行动”，开设“南昌经济110”企业投诉热线，建立服务业企业联席会议机制。建立用工信息对接机制、劳动力资源储备制度、用工劳务协作机制，解决企业用工难题；着力搭建企业与院校、院所、金融企业的联系平台，加强对企业管理咨询、创业辅导、企业上市前期等方面服务。强化招商创新。联手海内外一流投资机构，拓宽完善产业基金平台，让专业的人做专业的事，不断提高招商效率。加强重大项目审批“一站式”、项目建设“一条龙”、企业投产“管家式”三个服务体系建设，形成严密完整的招商引资服务体系。增强生态在招商引资中的吸引作用，将绿水青山变为金山银山。

（三）主动适应城镇化发展新理念，促进城市品位提升

坚持新老联动、两岸共辉，深入推进新城建设和旧城改造三年强攻计划，以新城发展带动老城改造，以老城改造支撑新城发展。坚持协调互进、城乡一体，构建城乡一体化新格局，推动新型城镇化科学有序发展。

一是遵循“旧城复兴”理念，完善城市规划。坚持城市功能新增与重构有机统一，全面弥补功能缺失、纠正功能错位；坚持空间改造和精神重塑有机统一，积极顺应城市自然肌理和市民生活需求；坚持重点突破与统筹推进有机统一，激发城市活力与实力、生态与文化的全面振兴。重点是尽快启动城市总体规划修编，做好对上一轮城市总体规划的评估；抓好旧城区街区整体提升改造规划、地铁以及地铁地上物业和地下空间开发利用规划；做好城关镇、特色镇及中心村、示范村的总体规划或单项规划，提前考虑扬子洲规划。

二是加大城市建设力度，强健城市筋骨。加快推进新城建设，全面完成九龙湖新城起步区范围拆迁，基本建成起步区市政路网框架，加快推进九龙湖湖体综合整治工程、隧道工程及景观绿化工程；加快九龙湖片区公共配套建设，推进220万平米动迁安置大社区建设。加快轨道交通和“十横十纵”快速路网建设，确保轨道交通1号线通车，加快2、3号线建设，启动4号线前期工作；确保朝阳大桥、象湖隧道、九洲高架按期通车，加快南外环高速公路、前湖大道快速路、天祥大道南延、昌西大道、志敏大道等快速路网建设，加快红谷隧道建设，推进昌九大道、105及320国道一级公路建设。启动火车站东广场改造工程（二期）建设，加快朝阳新城基础设施以及临空经济区基础设施建设，实施建设西路西延等断头路打通工程。

三是加快公共服务配套，优化城市功能。大力实施旧城区连线成片改造提升计划，在环境、功能和业态上做好文章。完成主城区400万平米的棚改征迁任务，重点推进十字街北街、二七路沿线等旧城（棚户区）改造项目。加快建设还建安置房小区，重点做好万寿宫历史文化街区改造建设。适时启动第三轮“拆违拆临、建绿透绿”工作，加强对“双拆”还路于民、还绿于民建设。巩固“畅通省城”工作成果，开发地下停车空间，多种方式缓解老城区“停车难”问题。大力推进“公交都市”创建工作，加大旧城管网改造力度。深入开展国家智慧城市试点。

四是坚持重心下移、面向市场，加强城市管理。围绕“干干净净、漂漂亮亮、井然有序、和谐宜居”目标，扎实开展“城市管理精细年”活动。着力完善商业特色街网络体系。深入开展“治脏治乱”工作，大力开展“五车”、“六乱”整治活动。深化城市管理体制改革，启动垃圾分类试点工作，重点探索推进市政养护、路灯管养的市场化。推进城市管理工作重心下移，健全街道综合执法队伍；强化属地管理，严格城市管理规范标准，加强和创新城市管理监督考核。

五是强化规范、突出重点，提升村镇建管水平。完善村民建房管理长效机制，规范村民建房行为。全面完成新农村建设任务，继续实施试点特色小镇、“五位一体”综合示范村建设，切实加强农村社会综合治理，改善农村公共服务。

（四）主动适应全面推进依法治市新常态，全面构筑发展环境核心竞争力

环境是发展的“气候”，企业是“迁徙的候鸟”，哪里气候适宜就会迁徙到哪里。必须围绕全面推进依法治市要求，构建一流发展环境。

一是努力打造公平正义的法治环境。着力建设平安南昌。严厉打击非法阻工、强揽工程、强买强卖等违法行为；以“两抢一盗”等侵财案件为重点，组织开展专项打击行动。强化矛盾纠纷化解。加强人民调解队伍建设，落实人民调解以奖代补机制，推动建立行业性、专业性人民调解组织。全面理顺信访秩序，落实信访联席会议制度，大力倡导网上信访。严格落实社会稳定风险评估。加快构筑社会诚信。加快诚信体系平台建设，建立健全信用信息基础数据库，提供信用信息服务。着力构建守信激励和失信惩戒机制，建立诚信红黑名单制度，努力营造诚实守信的社会氛围。

二是努力打造廉洁高效的政务环境。坚持依法行政。深入推进政府机构改革和职能转变，建立政府权力清单、责任清单和负面清单。健全依法决策机制，自觉接受人大的法律监督和政协的民主监督，建立行政机关内部重大决策合法性审查机制和重大决策终身责任追究制度及责任倒查机制。坚持高效诚信。试行新的建设项目审批流程，推进并联审批、网上审批，提高行政审批效率。消除行政审批灰色地带，坚决整治中介乱象。强化政府诚信，政府作出的承诺必须一一兑现。坚持阳光廉洁。以公开为原则，以不公开为例外，深入推进政务公开。深入开展作风转变、环境优化专项整治和监督检查。建立“啄木鸟暗访、政风行风热线曝光、责任追究”联动机制，对损害发展环境的人和事，发现一起，查处一起。以开展“两个责任实施年”为契机，建立健全反“四风”的长效机制，强化执纪监督。严格落实中央八项规定等廉洁从政各项规定，全面加强反腐倡廉基础制度建设，加大惩治腐败和纪律约束力度，坚决遏制腐败滋生。努力建设廉洁政府。

三是努力打造文明包容的人文环境。重描底色，践行核心价值观。在全市范围大力宣传社会主义核心价值观，积极开展涵养核心价值观的实践活动，使核心价值观渗透到经济社会各领域。唱响“八一”，激活红色基因。大力弘扬八一精神，认真筹备2017年建军90周年活动，创建全国双拥模范城。擦亮名片，彰显城市精神。以各类志愿者活动、相关专项整治为重点，培育彰显“大气开放、诚信图强”的城市精神，驱除人文环境的“雾霾”，维护精神家园的“蓝天”。

四是努力打造山清水秀的生态环境。全面加强生态文明建设，加大“治水”、“治气”、“治土”力度。做特水品牌。围绕“鄱湖明珠·中国水都”建设和全国水生态文明城市试点，加强重点水域整治，大力实施乌沙河治理、幸福渠综合治理等一批重点项目；深入推进梅湖景区改造提升和大象湖景区建设，加快规划建设赣江风光带、凤凰洲市民公园等重点项目；积极配合省里做好赣抚尾闾综合治理工程。做优绿生态。围绕创建国家生态园林城市、国家森林城市，积极开展城市生态敏感区园林修复，加快推进植物园建设，积极筹备省第四届花卉园艺博览会，启动申办第十二届中国国际园林博览会。做足美文章。全面整治农村水环境、生产生活垃圾和农业面源污染，切实改善农村生态环境。力争全年空气质量优良率达到70%以上，集中式饮用水源地水质达标率稳定在100%，主要河流水质达标率大于90%。

（五）主动适应民众过上美好生活新期待，全面推进社会民生事业发展

坚持“发展为民、发展惠民，急民所急、解民所忧”，打造更均等、更全面的公共服务网，织牢保基本、兜底线的民生安全网，让人民群众过上更加舒适、更加美好的生活。

一是服务均等。制定实施第二轮学前教育三年建设行动计划，增加公办和普惠性幼儿园数量。继续实施农村中小学标准化建设工程，完成校舍和教学装备改造项目建设。推进学校网点布局调整、名校领衔集团化、学区化办学，提高优质教育覆盖率。加快普通高中和职业教育发展。提升市属高校建设水平。加快建设区域人口健康信息平台，推行家庭医生（乡村医生）签约服务模式；优化医疗卫生服务流程，不断提升群众对医疗服务的满意度。推进城区“三个中心”标准化建设，确保完成三分之二以上进度。推进“城区公共图书馆联盟服务”带动工程，探索打造全市公共数字文化平台；推进农家书屋出版物更新、免费为农民送戏、自办文体活动等各项工作。

二是守住底线。重点加强社会救助、安置优抚工作。适时适度提高城乡低保保障标准和财政补差水平，提高农村五保户供养标准，提高企业军转干部、重点优抚对象和参战参试退役人员生活补助标准。深入开展免费救治，帮助残疾人康复和改善生活条件，支持儿童福利机构建设，关爱农村留守儿童，启动国家司法救助工作。科学开展扶贫开发和救灾救济工作。继续推进100个重点村扶贫开发工作。安排救灾救济专项资金，确保灾害发生后群众基本生活有保障，生产得到快恢复。

三是突出重点。全面扩大就业，全市城镇新增就业6.87万人，就业困难人员就业0.69万人，确保有就业能力的零就业家庭至少有一人就业；新增转移农村劳动力3.3万人。提高保障水平，全面完成城乡居民医疗保险整合；提高城乡养老保险补助标准和企业退休人员基本养老金水平；扩大“五险”覆盖面。强化住房保障，保障性住房续建项目完成投资30%，新建项目完成投资20%；加快困难群众危房和国有林场危旧房改造。方便居民生活，加大“煤改气”项目开发力度，发展民用户86000户；推进34家农贸市场的升级改造；建设社区连锁便利店100家，改造路灯6601盏、公厕199座。完善质量安全监管体系，确保食品安全。再解决22.27万农村人口饮水安全问题。

四是完善制度。推进新农保和城乡居保制度合并实施，完善社会保险区域对接机制；建立健全企业退休人员基本养老金、城乡居民基础养老金、城乡低保、重点优抚对象生活补助增长机制，建立健全特别救助制度。

各位代表，站在新的历史起点，面对省委、省政府和市委的新要求以及全市人民的新期待，我们深感责任重大、使命光荣。让我们更加紧密地团结在以习近平同志为总书记的党中央周围，在省委、省政府和市委的坚强领导下，抢抓机遇，务实奋进，为打造带动全省发展的核心增长极，加速“发展升级、小康提速、绿色崛起、实干兴赣”的进程而不懈奋斗!
